# Use of emergency medical services in Liberia: a retrospective review of a novel EMS system

**DOI:** 10.1186/s12245-026-01223-z

**Published:** 2026-04-24

**Authors:** Prem Menon, Mark Luke, Timothy Moran, Shea Sparks, Anna Yaffee, Elizabeth Peacock, Irfan Husain

**Affiliations:** 1 Department of Emergency Medicine, Martha’s Vineyard Hospital, Oak Bluffs, USA; 2 Emergency Medical Response, Liberia Ministry of Health, Monrovia, Liberia; 3 Department of Emergency Medicine, Emory University, Atlanta, USA; 4 Emergency Medicine of Idaho, Boise, USA

**Keywords:** Liberia, EMS, Prehospital, Obstetric care, Trauma care, Emergency care

## Abstract

**Background:**

The concept of prehospital care in Africa is still evolving, with only one-third of African countries having an established Emergency Medical Services (EMS) system. In 2019, the Ministry of Health of the Republic of Liberia created its first division of EMS. This study aims to characterize use of EMS in Liberia over a period of 22 months after its inception in order to guide system improvement and resource allocation.

**Methods:**

This is a retrospective review of the monthly EMS dispatch report summaries within Liberia from July 2020 to April 2022. A descriptive analysis was conducted to examine call characteristics, chief complaints, geographic call distribution, and patient destinations.

**Results:**

During the study period, 4,494 EMS calls were recorded, with 89.3% resulting in transport. The majority of patients were female (59.9%, *n* = 2694) and aged 16-35 years (52.1%, *n* = 2343). Most calls originated from the Commonwealth and Central Monrovia health districts, both located in the most populous county, Montserrado. John F. Kennedy (JFK) Hospital, located in the capital city of Monrovia, received the highest volume of patients (42.6%, *n* = 1708). Obstetric complaints, particularly labor, were the most common (23.8%, *n* = 955), followed by infectious disease (17.4%, *n* = 700) and road traffic accidents (7.6%, *n* = 306).

**Conclusion:**

This study presents the first review of data from Liberia’s national EMS system. The high prevalence of obstetric, infectious disease, and road traffic accident calls reflects the high burden of time-sensitive conditions faced by low- and middle-income countries (LMICs) and underscores the need to support access to emergency care, in which prehospital care is a key component. The findings further support the importance of targeted EMS training, protocol development, and resource allocation for highfrequency complaints.

## Background

Emergency medical services (EMS) is a vital part of the healthcare infrastructure for both developed countries and low- and middle-income countries (LMICs) due to the high disease burden of time-sensitive injuries and illnesses. It is estimated that up to 45% of deaths and 35% of disability-adjusted life years in LMICs could be potentially addressed by strengthening public access to the emergency care continuum through the development of EMS systems [1–3]. The World Health Organization’s (WHO) “Emergency Care System Framework” [4] underscores the critical role of EMS in providing on-scene response and facilitating transport from the field to healthcare facilities, contributing to the strengthening of national emergency care systems.

In 2017, a comprehensive assessment of EMS systems across Africa revealed significant disparities in availability and quality [2]. Approximately one-third of African nations have established EMS systems, yet less than 9% of the continent’s population has access to these services. The primary prehospital emergencies involve injuries and obstetric complications. Most existing EMS systems are government-operated, predominantly offer Basic Life Support (BLS), and function on a fee-for-service basis [2, 3]. At the time of the 2012 assessment, Liberia population was part of the 91% without access to an EMS system.

Liberia has endured colonization, political collapse, civil wars, the Ebola epidemic, and the COVID-19 pandemic, leaving widespread poverty and a fragile healthcare system [5, 6]. Most of the population resides in the capital city of Monrovia, home to the tertiary referral hospital. Following the Second Liberian Civil War, U.S. institutions aided in rebuilding medical care, including emergency medicine training, but progress was disrupted by the 2014 Ebola outbreak, which reduced healthcare services by 50% [7]. Efforts to improve emergency care have been limited, with the most recent data from 2012 highlighting severe deficiencies in pre-hospital care. In order to address this deficiency, in 2019 the Ministry of Health launched a national EMS system.

As Liberia develops an emergency care infrastructure, understanding the use of EMS within the broader emergency care framework is essential. Currently, no published literature exists on the use of Liberia’s new national EMS system. This study presents an initial analysis of data from the national EMS system through a retrospective review of monthly EMS dispatch reports, providing insights into EMS use across the country. Findings from this study will inform resource allocation and deployment strategies, as well as guide prehospital provider training and protocol development.

## Methods

### Study setting

The national-run EMS system covers all of Liberia and is accessible by calling a toll-free number 24 h a day. Ambulance service is provided by government funding at no cost to the patient through government funding, but some associated expenses, such as medications, medical supplies, or fuel, may result in additional charges. The EMS system consists of 62 ambulances, all of which are basic life support (BLS) units, and are staffed by 48 BLS-level trained prehospital personnel who undergo emergency medical technician (EMT) refresher training updates on a quarterly basis. The ambulance crews typically include 2 EMTs. The remainder of the 350 trained personnel involved in EMS operations include additional support staff, nurses, and dispatch agents. While the center of operations is at John F. Kennedy (JFK) hospital, ambulances are strategically placed at various health care facilities to improve access and response times.

### Data collection

We conducted a retrospective review of aggregated monthly dispatch reports for all EMS calls and transports managed by the national Liberia EMS system over 22 months from July 2020 to April 2022. The monthly dispatch reports included data on the number of calls received by dispatch, the number of patients transported, patient age and gender, chief complaints, receiving facilities, dispatch locations, and responding ambulances. These de-identified reports were regularly compiled for quality assurance purposes from individual patient care reports (PCRs) from all 15 counties in Liberia (*N* = 4,494). Due to recording inconsistencies in earlier dispatch reports, data prior to July 2020 were excluded from analysis. Consistent documentation began starting July 2020 and thus data was used starting from this time. This study was deemed exempted from obtaining ethical approval by the Emory University Institutional Review Board.

### Data analysis

We performed a descriptive analysis of call characteristics, chief complaints, geographic call distribution, and patient destinations. Characteristics were described using frequencies and percentages. Chief complaints from the monthly reports were sub-categorized into obstetric (OB), trauma, or medical-related categories. We further classified OB-related complaints based on the stages of pregnancy, trauma-related complaints by injury type and mechanism, and medical complaints using a systems-based approach when feasible. Many medical complaints were initially labeled as “Sick Person.” Due to the high volume and impracticality of reviewing each “Sick Person” PCR, this category was retained as a standalone classification. All data analysis was conducted in R (v4.1; R Core Team).

## Results

### Call characteristics

Over the study period, a total of 4,494 calls were recorded, of which 89.3% (*n* = 4,014) resulted in a patient transport and 10.7% (*n* = 480) were not transported. Among those not transported, the most common reasons were cancellation (41.9%, *n* = 201), treatment on scene (23.8%, *n* = 114), and patient death prior to arrival (15.8%, *n* = 76). Of the total calls recorded, the majority of patients were female (59.9%, *n* = 2694), and the most represented age group was 16–35 years (52.1%, *n* = 2343), followed by 36–45 years (19%, *n* = 855) and those over 45 (18.4%, *n* = 826) (Table 1).

**Table 1:** Call characteristics - the majority of calls were transported to a healthcare facility with most calls being from female patients and young adults (16–35 years of age)

Characteristic	*N*	%
**Total Calls**	4494	-
**Total Transported**	4014	89.3
**Total Not Transported**	480	10.7
**Sex**		
Male	1800	40.1
Female	2694	59.9
**Age**		
0–15	470	10.5
16–35	2343	52.1
36–45	855	19
> 45	826	18.4
**Reason for Not Transporting**		
Canceled	201	41.9
Dead	76	15.8
Lack of Fuel	11	2.3
Private Vehicle	48	10
Refused	30	6.3
Treated on Scene	114	23.8

### Geographic call distribution

Call volume varied by health district, with the highest number of calls originating from Commonwealth Health District (34.6%, *n* = 1388) and Central Monrovia (18.6%, *n* = 747), both located in the most populous county of Montserrado. Montserrado County includes the capital city of Monrovia (Table 2).

**Table 2:** Call volume by health district - the majority of calls originated from Montserrado County, which includes the capital city of Monrovia

District	*N*	%
Bushrod Island Health district	373	9.3
Caresburg Health district	101	2.5
Central Monrovia Health district	747	18.6
Commonwealth Health district	1388	34.6
Other (Margibi − 16, Grand Bassa 1, Bong 1.)	943	23.5
Somalia Drive Health district	387	9.6
St. Paul Health district	52	1.3
Todee district	23	0.6

### Patient destinations

While there are more than 100 unique health centers to which patients were transported, the three most common receiving hospitals were all located in the capital city of Monrovia. The most common receiving hospital is John F. Kennedy (JFK) Hospital (42.6%, *n* = 1708), the largest tertiary care center of Liberia (Table 3).

**Table 3:** Distribution of Patients between Receiving Facilities - the most common receiving hospital was JFK Hospital, the largest tertiary care center in Liberia

Receiving Facility	*N*	%
JFK Hospital	1708	42.6
Star Base	276	6.9
ELWA	274	6.8
14th Military Hospital	213	5.3
JDJ Hospital	143	3.6
Redemption	127	3.2
Catholic Hospital	77	1.9
Kolahun Hospital	70	1.7
MSF	65	1.6
Union Central Treatment Unit	57	1.4
Home	71	1.8
All Others	933	23.2

JKF Hospital - John F. Kennedy Hospital; ELWA - Eternal Love Winning Africa Hospital; JDJ Hospital - James N. Davis Jr. Memorial Hospital; MSF - Médecins Sans Frontières

### Chief complaints

Chief complaints were categorized into obstetric (OB), medical, trauma, and surgical presentations. OB-related calls accounted for a substantial portion of the caseload (35.5% of total transported), with labor comprising 23.8% (*n* = 955) of total cases, followed by antepartum (8.7%, *n* = 348) and postpartum issues (3.0%, *n* = 121). Among medical complaints (accounting for 52.8% of the dataset), infectious diseases were most frequent (17.4%, *n* = 700), followed by nonspecific “sick person” (4.5%, *n* = 182), cardiovascular (4.1%, *n* = 163), respiratory (3.3%, *n* = 134), and neurologic (3.1%, *n* = 126) issues. The prevalence of calls related to infectious disease highlights the significant burden and critical role EMS systems play in connecting patients to care and in disease surveillance.

**Chart 1:**
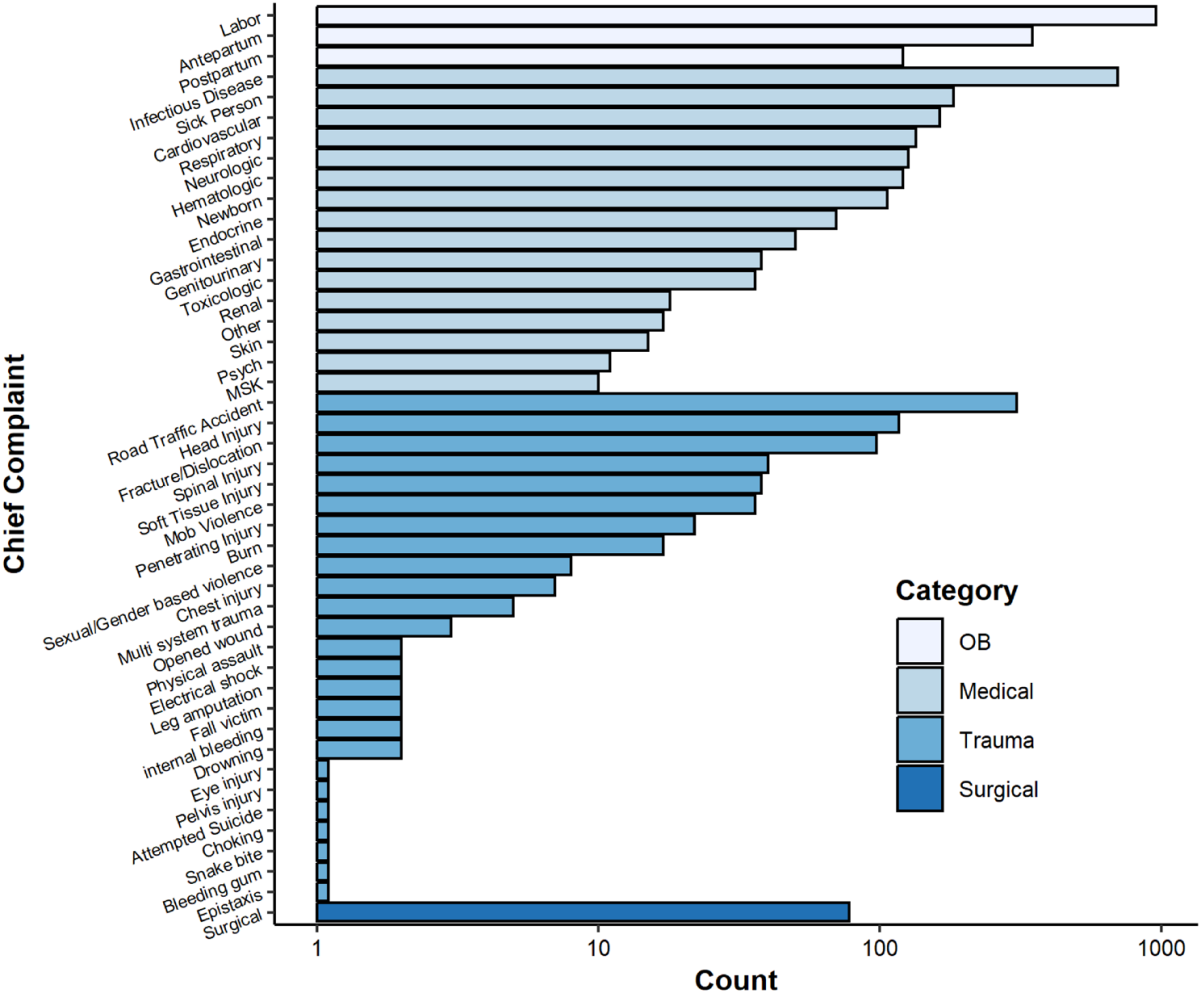
Chief complaint distribution visualized

Trauma-related cases made up a significant proportion of the dataset (17.3%, *n* = 716 of total), with road traffic accidents (RTAs) being the most common at 7.6% (*n* = 306 of total cases, 42.8% within trauma). Again, the burden of time sensitive injuries and frequent occurrence of RTAs demonstrates the potential impact of robust EMS systems in improving access to care. Other trauma presentations included head injuries (2.9%, *n* = 117), fractures/dislocations (2.4%, *n* = 97), and spinal injuries (1.0%, *n* = 40). Surgical presentations are defined as patients presenting with post-operative related complaints. Surgical complaints were infrequent, comprising just 1.9% (*n* = 78) of the sample (Table 4) (Chart 1).

**Table 4:** Chief complaint distribution - obstetric concerns represented the largest proportion of EMS calls, followed by medical and trauma-related complaints

Category	Chief Complaint	Count	Percent of Sample	Percent within Category
OB	Labor	955	23.8	67.1
OB	Antepartum	348	8.7	24.4
OB	Postpartum	121	3.0	8.5
Medical	Infectious Disease	700	17.4	39.0
Medical	Sick Person	182	4.5	10.1
Medical	Cardiovascular	163	4.1	9.1
Medical	Respiratory	134	3.3	7.5
Medical	Neurologic	126	3.1	7.0
Medical	Hematologic	121	3.0	6.7
Medical	Newborn	106	2.6	5.9
Medical	Endocrine	70	1.7	3.9
Medical	Gastrointestinal	50	1.2	2.8
Medical	Genitourinary	38	0.9	2.1
Medical	Toxicologic	36	0.9	2.0
Medical	Renal	18	0.4	1.0
Medical	Other	17	0.4	0.9
Medical	Skin	15	0.4	0.8
Medical	Psych	11	0.3	0.6
Medical	Musculoskeletal	10	0.2	0.6
Trauma	Road Traffic Accident	306	7.6	42.8
Trauma	Head Injury	117	2.9	16.4
Trauma	Fracture/Dislocation	97	2.4	13.6
Trauma	Spinal Injury	40	1.0	5.6
Trauma	Soft Tissue Injury	38	0.9	5.3
Trauma	Mob Violence	36	0.9	5.0
Trauma	Penetrating Injury	22	0.5	3.1
Trauma	Burn	17	0.4	2.4
Trauma	Sexual/Gender violence	8	0.2	1.1
Trauma	Chest injury	7	0.2	1.0
Trauma	Multi system trauma	5	0.1	0.7
Trauma	Opened wound	3	0.1	0.4
Trauma	Physical assault	2	< 0.1	0.3
Trauma	Electrical shock	2	< 0.1	0.3
Trauma	Leg amputation	2	< 0.1	0.3
Trauma	Fall victim	2	< 0.1	0.3
Trauma	internal bleeding	2	< 0.1	0.3
Trauma	Drowning	2	< 0.1	0.3
Trauma	Eye injury	1	< 0.1	0.1
Trauma	Pelvis injury	1	< 0.1	0.1
Trauma	Attempted Suicide	1	< 0.1	0.1
Trauma	Choking	1	< 0.1	0.1
Trauma	Snake bite	1	< 0.1	0.1
Trauma	Bleeding gum	1	< 0.1	0.1
Trauma	Epistaxis	1	< 0.1	0.1
Surgical	Surgical	78	1.9	100

## Discussion

The majority of EMS calls to the national Liberia EMS system during the reporting period were obstetric-related, followed by infectious disease and road traffic accidents. A high proportion of patients were transported, with most receiving hospitals located in Monrovia, the capital. These findings reflect the critical role EMS is playing in Liberia’s health system, by providing care for time-sensitive conditions and access to advanced care hospitals, like JFK Hospital.

The prominence of obstetric-related calls aligns with Liberia’s ongoing maternal health crisis, with one of the highest maternal mortality rates globally, 742 per 100,000 live births [8]. Although childbirth in rural health facilities and maternity waiting homes has increased [9, 10], many of these facilities lack emergency readiness and the means for rapid referral. Only 40% of rural health facilities have both a functional ambulance and a working mobile phone to initiate transfer [11]. In this context, EMS offers a vital link in the chain of survival for maternal emergencies, suggesting a potential shift in cultural norms and a growing reliance on EMS to access skilled care. Our data support this notion and underscore the need for obstetric-specific training and resource allocation within the EMS system.

Beyond obstetric emergencies, our findings show a high burden of trauma from road traffic accidents (RTAs) and infectious disease-related emergencies, conditions that are among the leading causes of death and disability in LMICs. Over 85% of global deaths and 90% of disability-adjusted life years (DALYs) from RTAs occur in LMICs [12]. These patterns mirror findings in other settings such as rural Uganda, where a newly implemented EMS system showed similar call volumes dominated by obstetric and trauma-related cases, underscoring the shared regional burden of time-sensitive conditions [13].

The Liberia EMS system appears to align with the WHO framework for emergency care, which emphasizes early recognition, prompt intervention, and rapid transport for time-sensitive conditions [14]. The majority of calls in our dataset were for obstetric, trauma, or infectious disease complaints, each associated with potentially preventable morbidity and mortality when care is delayed. The fact that EMS transported most patients to tertiary facilities suggests that the system is functioning as a conduit to higher levels of care. However, whether this pattern reflects clinical necessity, geographic proximity to Monrovia-based hospitals, or referral decisions made from community health centers remains unclear. Future studies with more granular data on origin, acuity, and clinical outcomes are needed to disentangle these variables.

Infectious disease emergencies, especially in the context of recent public health crises like Ebola, also comprise a meaningful share of EMS call volume. Liberia, like many West African countries, continues to face periodic infectious disease outbreaks, and EMS systems can support timely response and containment efforts. The Africa CDC’s initiative to expand event-based surveillance systems [15] is a promising complement to EMS development. Strengthening EMS with data-informed protocols for infectious disease transport, personal protective equipment, and bio surveillance coordination will further enhance outbreak responsiveness [16, 17].

Nevertheless, several limitations must be acknowledged. As a retrospective analysis of patient care records, the data are subject to documentation errors, inconsistent protocols, and limited patient outcome tracking. Despite these constraints, the data can inform targeted EMS training, protocol development, and strategic ambulance placement. These interventions have the potential to reduce response times, enhance patient care, and ultimately optimize the impact of EMS in managing Liberia’s high-burden, time-sensitive conditions.

## Conclusion

This is the first review of EMS data from Liberia, aimed at understanding the early implementation and utilization of the national EMS system. The most common reasons for EMS activation included obstetric emergencies, infectious diseases, and road traffic-related trauma, with females and young adults (ages 16–35) representing the majority of users. These findings underscore the initial need for targeted provider education, protocol development, and strategic ambulance placement to appropriately care for these high-volume, time-sensitive conditions. The data emerging from Liberia’s national EMS system mirrors trends observed in other LMICs [1, 2, 12], with recent literature suggesting the development of EMS systems as becoming a public health priority [1, 3, 5, 7]. Future efforts should prioritize the development of dispatch systems, strategic ambulance locations for optimal coverage, and improved communication pathways with receiving facilities to enhance patient coordination and continuity of care. As the system continues to evolve and is guided by ongoing data collection and analysis, EMS will likely play an increasingly important role in public health and surveillance. Exploring how ambulance and personnel availability affects access to higher-level care could inform strategies to expand healthcare access nationwide and inform ongoing policy.

## Data Availability

The datasets generated and/or analyzed during the current study are not publicly available due to national data governance restrictions and institutional agreements with the Ministry of Health of the Republic of Liberia. Although the data were de-identified prior to analysis, public sharing is restricted. The data are available from the corresponding author on reasonable request and with permission of the Ministry of Health of the Republic of Liberia.

## Abbreviations

EMS: Emergency Medical Services
LMICs: Low–and Middle–Income Countries
WHO: World Health Organization
BLS: Basic Life Support
EMT: Emergency Medical Technician
PCR: Patient Care Report
IRB: Institutional Review Board
OB: Obstetric
RTA: Road Traffic Accident
DALYs: Disability–Adjusted Life Years
CDC: Centers for Disease Control and Prevention
JFK: John F. Kennedy Hospital
ELWA: Eternal Love Winning Africa Hospital
JDJ: James N. Davis Jr. Memorial Hospital
MSF: Médecins Sans Frontières
